# Pericardial Hernia After Pericardial Window: A Rare Case

**DOI:** 10.7759/cureus.70152

**Published:** 2024-09-25

**Authors:** Sandeep Maharajh, James Pilkington, Qutayba Almerie, Paul Goldsmith

**Affiliations:** 1 General Surgery, Manchester University NHS Foundation Trust, Manchester, GBR

**Keywords:** iatrogenic complication, mesh repair, pericardial effusion, pericardial hernia, pericardial-peritoneal window

## Abstract

Diaphragmatic hernias have classically been reported due to congenital birth defects and blunt or penetrating trauma. We present a rare case of an intrapericardial diaphragmatic hernia after left-sided pericardial window surgery for chronic pericardial effusions. A 59-year-old female with a background of systemic lupus erythematosus and recurrent pericardial effusions underwent subxiphoid placement of a pericardial-peritoneal window. Postoperatively, she reported exertional shortness of breath. Imaging revealed a diaphragmatic hernia in the pericardial cavity. Open adhesiolysis between the abdominal organs and the heart was performed via a thoracoabdominal approach followed by suture and mesh repair of the defect. The postoperative course was uneventful. Pericardial hernias are rare, with few published cases. Their etiologies can be traumatic, iatrogenic, or congenital, with variable, non-specific symptoms that may occur at any time after the inciting event. With the potential for significant cardiac compromise, clinicians should be aware of this rare diagnosis, and surgical repair must be prioritized. This case documents the successful surgical management of this rare complication. Careful multidisciplinary planning is essential for surgical repair and should be tailored to patient-specific factors.

## Introduction

Pericardial hernias are rare with potentially life-threatening complications. Clinical presentation varies, with diagnoses made incidentally in asymptomatic patients to patients with severe cardiorespiratory distress and cardiac tamponade [1]. In 1903, De Cardinal et al. were the first to publish a case of congenital pericardial hernia containing a transverse colon, and in 1910, Keith published the first pericardial hernia of traumatic etiology on examination of two cadaveric specimens [2,3]. This report highlights an unusual case of pericardial hernia after the pericardio-peritoneal window for the management of a chronic pleural effusion, its radiological findings, and surgical management.

## Case presentation

**Figure 1:**
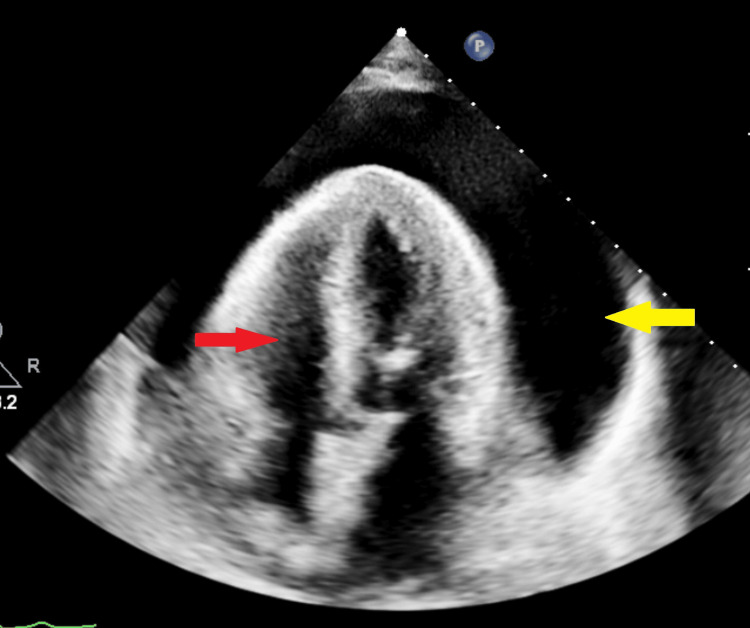
Echocardiogram showing apical four chamber view with large circumferential pericardial effusion (yellow arrow) and right ventricle (red arrow) for reference

A 59-year-old female presented to the cardiology clinic with worsening exertional dyspnea and no acute inflammatory signs or symptoms. Past medical history included hypertension, hyperlipidemia, chronic microcytic anemia, and a 20-pack-year history of cigarette use. The echocardiogram revealed a small pericardial effusion, and the NT-proBNP level was 642 pg/ml (normal < 125 pg/ml). Complaints of lethargy and weakness prompted rheumatology consultation, and serological investigation confirmed the diagnosis of systemic lupus erythematosus. Management was started with oral prednisolone and daily mycophenolate mofetil. There was no evidence of right heart strain or cardiac tamponade, so serial six-monthly echocardiograms were performed. Despite initial improvement of symptoms on medical therapy, symptoms recurred after two years, and an echocardiogram revealed a 3.8 cm pericardial effusion (Figure 1). Pericardiocentesis was performed with the relief of symptoms.

**Figure 2:**
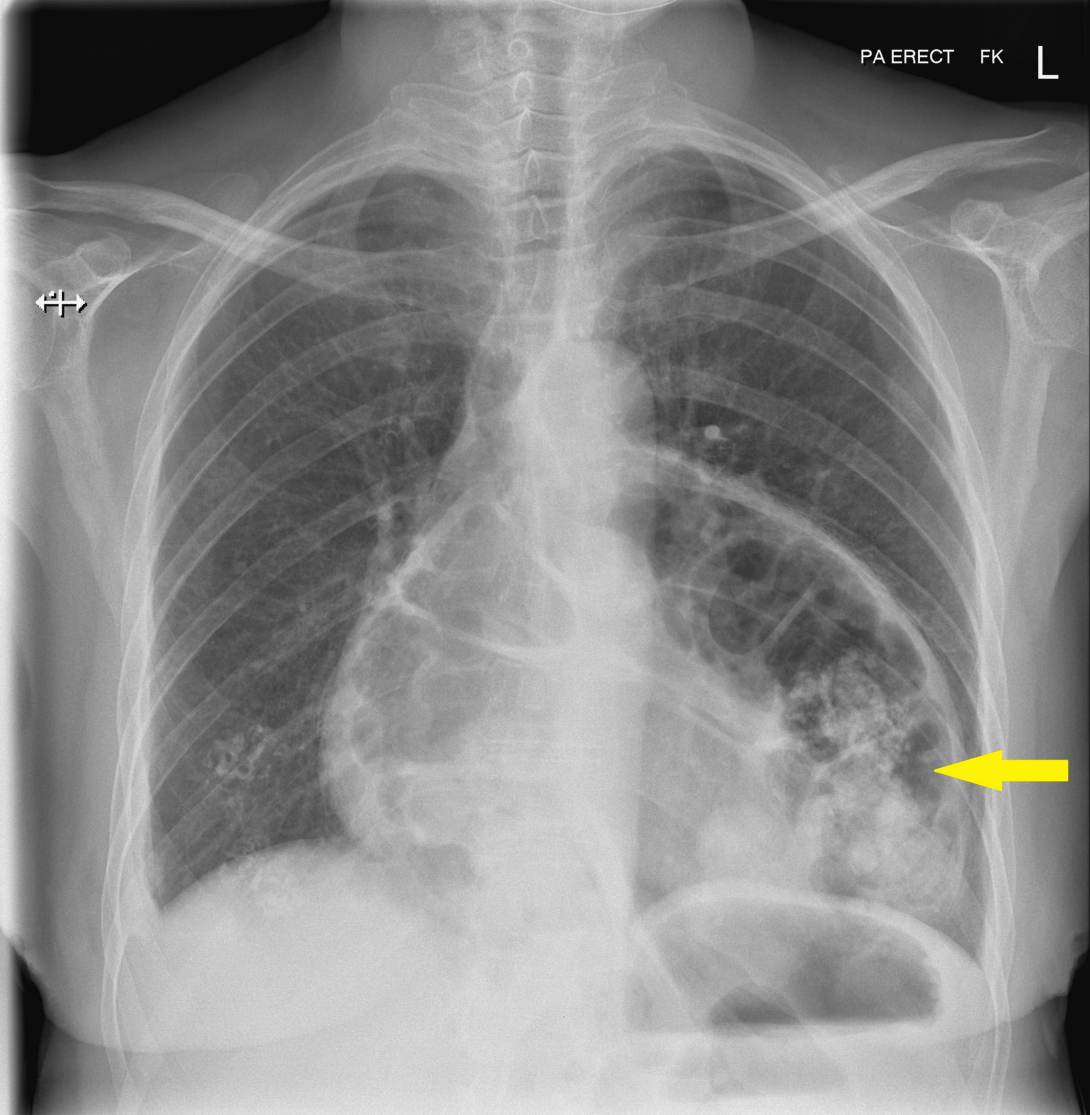
Erect chest X-ray showing large bowel loops within the mediastinum (yellow arrow) and clear lung fields

**Figure 3:**
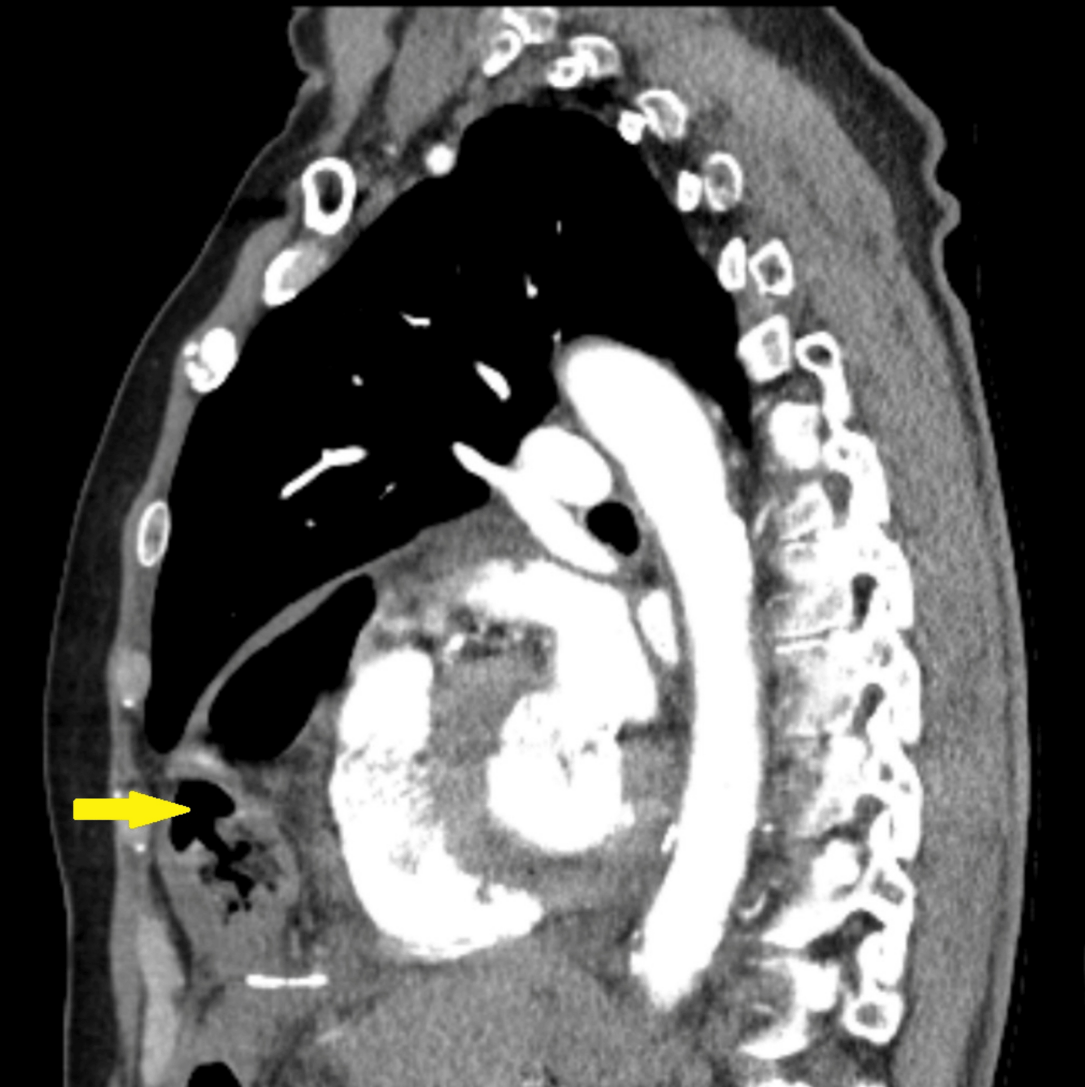
CT sagittal view showing transverse colon (yellow arrow) anterior to the heart within the pericardial sac

**Figure 4:**
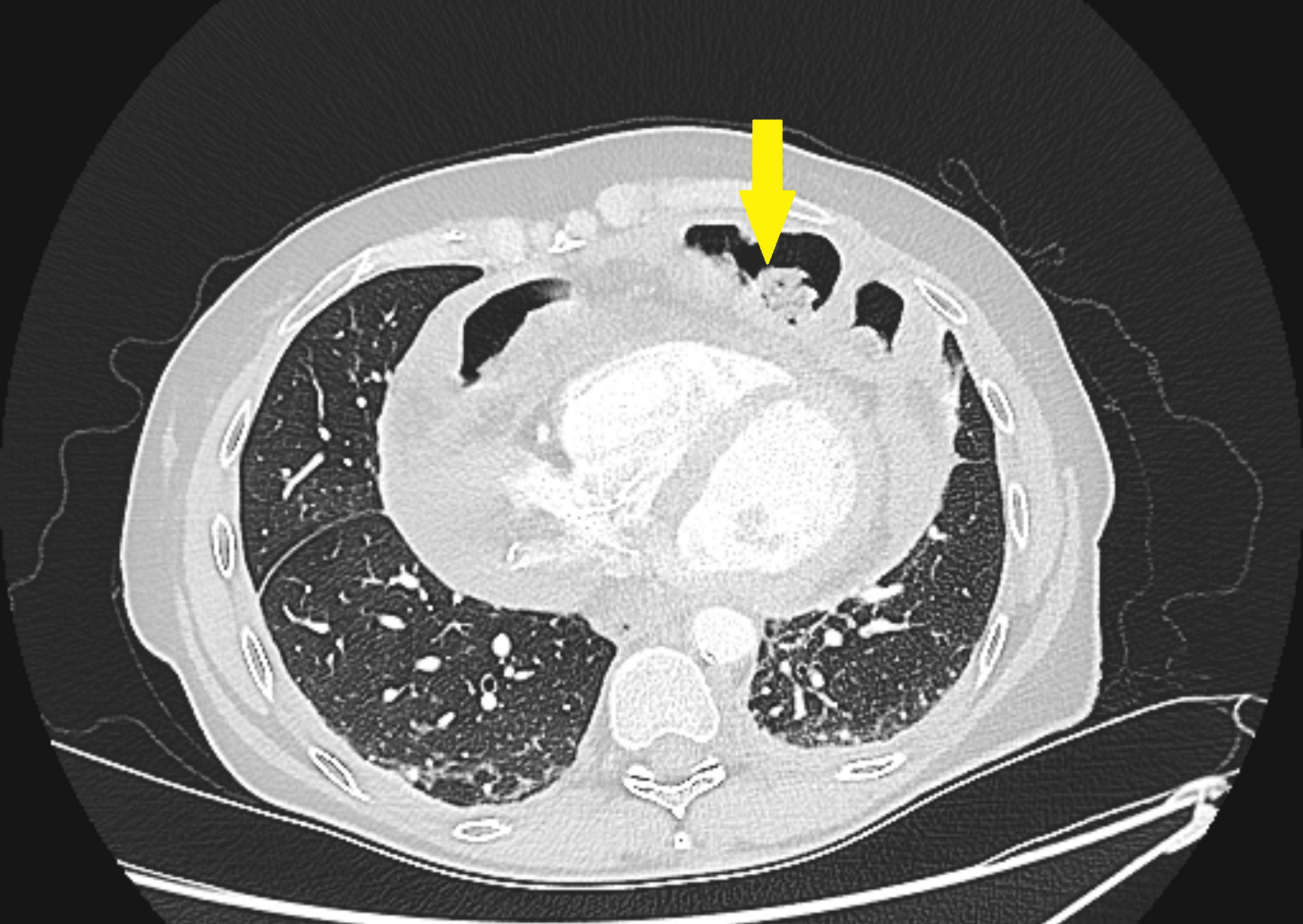
CT axial view showing transverse colon (yellow arrow) anterior to the heart within the pericardial sac

Six months later, there was re-accumulation of a large circumferential pericardial effusion. A pericardial window via a subxiphoid approach was made due to the recurrent nature of the effusion. Five months following this procedure, the patient reported worsening exertional dyspnea, epigastric and chest pains, intermittent non-productive cough, and palpitations. Curiously, the chest pain worsened with eating and there was mild relief with bowel movements. An echocardiogram was reported as poor views of the heart. Erect chest X-ray revealed a large bowel around the heart within the mediastinum (Figure 2). The diagnosis of a pericardial diaphragmatic hernia was then confirmed on computed tomography (Figures 3, 4).

**Figure 5:**
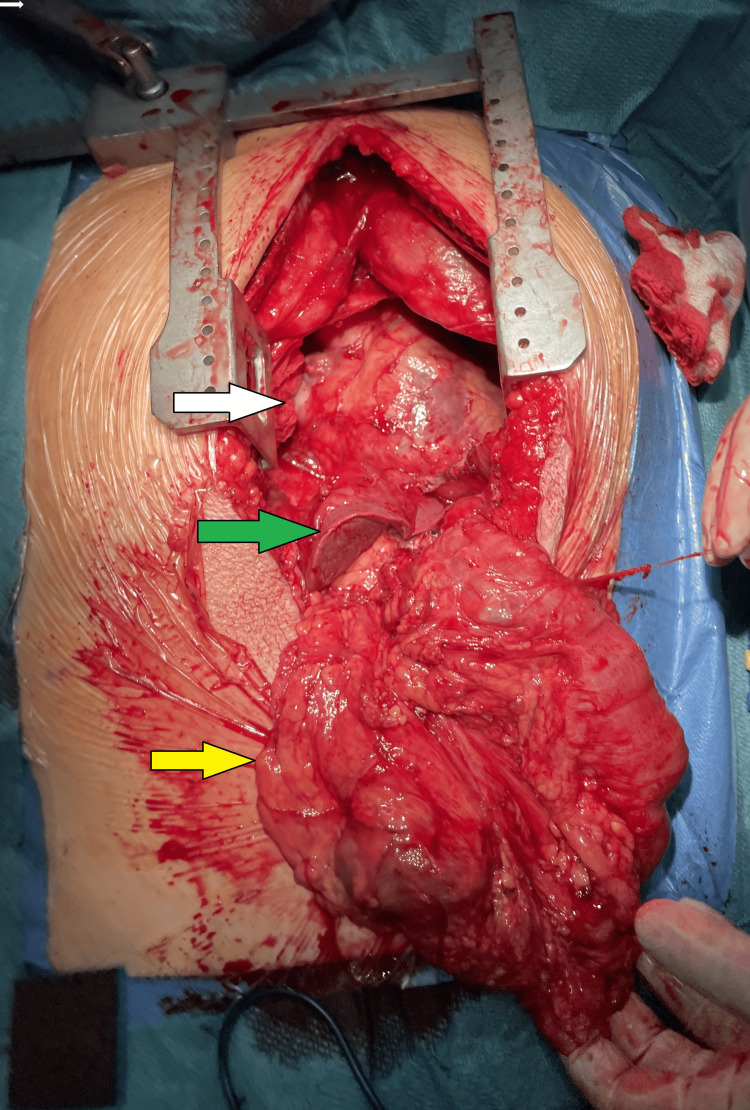
Midline thoracoabdominal incision showing anterior border of heart (white arrow), left lobe of liver (green arrow) and reflected transverse colon (yellow arrow) after adhesiolysis

After a multidisciplinary team meeting amongst cardiothoracic surgery, general surgery, cardiac anesthesiologists, and cardiology critical care, a decision was made to proceed with open surgical repair. With the patient supine, an initial upper abdominal midline incision was made. The transverse colon and the left lobe of the liver were visible herniating through a defect in the central tendon of the diaphragm. Adhesions prevented the reduction of the viscera back into the abdominal cavity. The incision was extended to a midline thoracotomy and the pericardium was opened to reveal flimsy adhesions attaching the transverse colon to the anterior cardiac wall. Adhesiolysis was performed as seen in Figure 5, and the colon was reduced into the abdominal cavity. Ligation of the falciform and coronal ligaments was done to improve access to the diaphragmatic defect. The left lobe of the liver was dissected off the diaphragm and returned to the peritoneal cavity.

The pericardium was closed, and the diaphragmatic defect was repaired with a continuous 0-ETHIBOND® EXCEL suture. A Bio-A® absorbable synthetic 9 cm x 15 cm mesh was shaped and sutured to the inferior aspect of the diaphragm with 0-ETHIBOND® EXCEL. The abdominal and thoracic walls were closed in layers. The patient was transferred to cardiac intensive care intubated, extubated on day two, and discharged well on day nine postoperatively.

**Figure 6:**
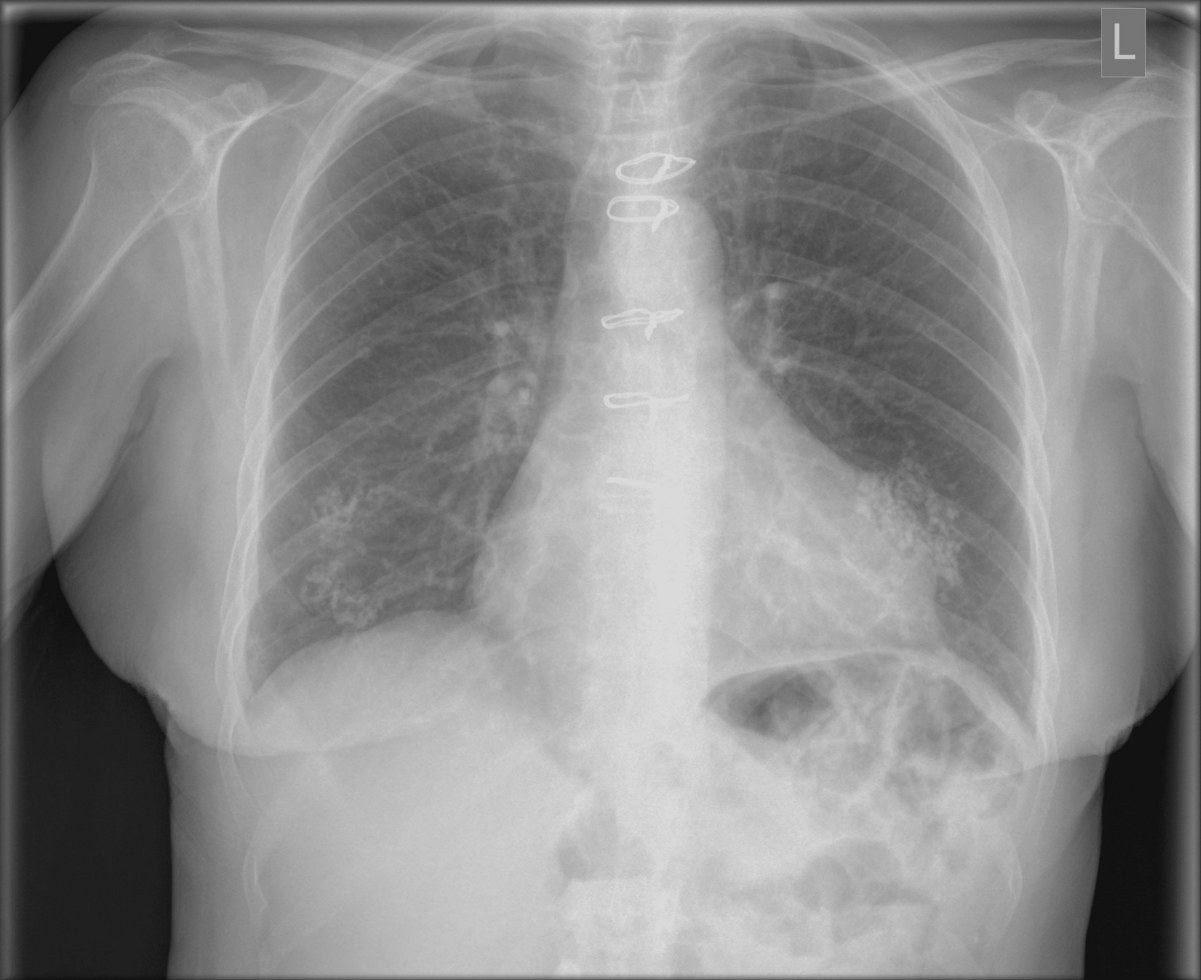
Erect chest X-ray showing no recurrence of hernia at six months postoperatively

The patient was asymptomatic with no recurrence of the hernia at six months postoperatively as documented by repeat erect chest X-ray (Figure 6). Serial echocardiograms, with a plan for repeat pericardiocentesis if needed, were performed.

## Discussion

A pericardial hernia is a rare, potentially life-threatening complication of a pericardio-peritoneal window. Symptomatology can be non-specific and can occur at varying intervals after the procedure. Additionally, the surgical approach and timing of repair should be tailored to the hemodynamic stability of the patient, chronicity of symptoms, and patient physical fitness. Furthermore, in cases of hernia due to iatrogenic injury during the treatment of existing cardiothoracic disease, surgical repair should factor in the additional long-term management of underlying illnesses.

Pericardial hernias occur from two underlying mechanisms. The outer fibrous pericardium is continuous with the central tendon of the diaphragm, and a congenital or iatrogenic defect here can act as a potential site of herniation into the pericardial sac. Furthermore, high-energy trauma to the abdomen that increases intra-abdominal pressures from 2-10 mm H_2_O to more than 1000 cm H_2_O can also cause herniation [4]. In their systematic review, Schizas et al. concluded that more than half of pericardial hernias are secondary to high energy, blunt force trauma, and 30% are attributable to iatrogenic injury, the most common procedure being subxiphoid pericardial window [5]. Pericardial hernias have been reported to comprise <1% of congenital diaphragmatic hernias and approximately 3% of diaphragmatic hernias due to trauma [6,7].

The decision for surgical management of a pericardial effusion should take into consideration the underlying etiology, response to medical management, hemodynamic stability of the patient, fitness, and long-term prognosis. The European Society of Cardiology (2015) states that underlying connective tissue disease is responsible for 5-15% of large pericardial effusions (i.e., >20 mm) and advises that pericardiocentesis or surgery is indicated for cardiac tamponade or large effusions not responsive to medical therapy and surgical drainage after pericardiocentesis should be considered in the event of recurrence [8,9].

Pericardial hernias can pose a diagnostic dilemma with a range of non-specific symptoms that may present at varying times after the inciting incident. Half of the patients with pericardial hernias from trauma are diagnosed immediately, but diagnosis six years after trauma due to a motor vehicle accident has been documented [1,10]. Abdominal pain and dyspnea are the two most common presenting complaints, but 10% of patients are asymptomatic [5]. Therefore, whether acutely unwell or presenting years after a potential inciting event, clinicians should be suspicious of pericardial hernias as a potential differential diagnosis in patients with non-specific cardiac or gastrointestinal symptoms and a history of abdominal trauma or relevant surgical history. Similarly, herniation should be definitively ruled out in patients managed for chronic pericardial effusions with surgical intervention due to the overlapping symptomology.

Surgical techniques for repair of pericardial hernias can be performed either laparoscopically or open and include abdominal, thoracic, and thoraco-abdominal approaches. In this case, the initial decision for an open abdominal approach was to gain adequate access to the diaphragmatic defect for repair while avoiding the potential cardio-respiratory compromise and complicated recovery associated with a thoracic approach, especially in an immunocompromised elderly patient with an inflammatory autoimmune connective tissue disease and smoking history. Extension of the incision to an open thoracotomy was needed to facilitate adhesiolysis between the heart and herniated colon. Similarly, Sharma recommends an abdominal approach for acute cases where rapid access to the diaphragm is needed but a thoracic approach to allow for lysis of adhesions more likely to be formed in chronic cases [11].

Primary suture repair of diaphragmatic defect, bolstered by absorbable synthetic mesh, was done in this case. Specific guidelines on the repair of pericardial hernias are limited, but in hiatal hernia repair, use of mesh reduces the risk of recurrence compared to suture repair alone [12]. An isolated case describing the use of a non-absorbable, non-covered macro-porous mesh without suture repair has also been reported to repair the defect while allowing continued drainage of the effusion [13]. This approach was used in a patient with a neoplastic pericardial effusion with a high risk of re-accumulation, a need for continued drainage, and difficulty with tissue approximation due to tension. Given the rarity of this diagnosis and range of surgical approaches, the anatomy of the defect, duration of symptoms, and clinician experience should guide the technique of surgical repair.

Pericardial hernias are rare, and there is a lack of literature on management approaches. The method of surgical repair should therefore be adapted specifically to each case. Patient comorbidities, fitness for surgery, likelihood of recurrence, and long-term prognosis should always be considered when deciding on suitable surgical management.

## Conclusions

The authors of this study report a complications from the pericardial window for the management of a chronic, large pericardial effusion. While pericardial hernias are rare, clinical suspicion should remain high in patients presenting with recurrence of symptoms, especially after surgical management of effusion. Surgical repair is advised, and the chosen approach and technique should be decided as a multidisciplinary team alongside patient counseling.
